# Twin pregnancy with complete hydatidiform mole and coexisting fetus following ovulation induction with a non-prescribed clomiphene citrate regimen: a case report

**DOI:** 10.1186/1752-1947-6-95

**Published:** 2012-04-03

**Authors:** Sherif Abd-Elkarim Mohammed Shazly, Mohammed Khairy Ali, Ahmed Yahia Abdel Badee, Abu-bakr Abbas Alsokkary, Mostafa Mohammed Khodary, Nehal Abd-Elkarim Mostafa

**Affiliations:** 1 Woman's Health Center, Assiut University, Assiut, Egypt; 2 Clinical Pathology Department, Assiut University, Assiut, Egypt

## Abstract

**Introduction:**

Twin pregnancy with complete hydatidiform mole represents a very rare obstetric problem. Management of such cases is always problematic because the possibility of fetal survival should always be weighed against the risk of complications of molar pregnancy.

**Case presentation:**

A 34-year-old Caucasian woman presented to our center with mild vaginal bleeding. Our patient was 16 weeks pregnant after a seven-year period of primary infertility. She became pregnant following a non-prescribed regimen of clomiphene citrate extending from the second day to the 13th day of her last cycle. A transabdominal ultrasound examination revealed a twin pregnancy with complete hydatidiform mole and a coexisting fetus. Serum β human chorionic gonadotropin was falsely low as identified by serial dilution of the sample (the 'hook effect'). Our patient refused termination of pregnancy and she was hospitalized for strict observation and follow-up. Unfortunately, she developed an attack of severe vaginal bleeding and a hysterotomy was performed. The fetus died shortly after birth.

**Conclusions:**

Twin pregnancy with complete hydatidiform mole represents a matter of controversy. We suggest that conservation should always be considered whenever tertiary care services and strict observation are available.

## Introduction

Twin pregnancy with complete hydatidiform mole and coexisting fetus is rarely seen during clinic practice. The incidence ranges from 1 in 20,000 to 1 in 100,000 pregnancies [1]. Diagnosis in such cases can be simply made by obstetric ultrasound examination but the decision whether to conserve or not is always problematic. Traditionally, termination of pregnancy was indicated to avoid the unacceptable risk of complications of complete molar pregnancy such as early onset pre-eclampsia, thyrotoxicosis and increased risk of persistent trophoblastic disease [2]. However, complete hydatidiform mole is possibly associated with advanced maternal age and the use of assisted reproductive techniques, and this reflects how difficult the decision of termination is for such couples [3]. Our case report represents one such patient who conceived after a relatively long period of infertility and repeated medical trials for conception. In this article, we report a case of second trimester twin pregnancy with a huge complete hydatidiform mole that was managed conservatively according to our patient's request and circumstances. We also discuss the falsely low β human chorionic gonadotropin (hCG) results that were revealed during follow-up in what is known as the 'hook effect'. The hook effect is another interesting phenomenon that should be considered in patients with complete molar pregnancy and low serum β-hCG.

## Case presentation

A 34-year-old Caucasian woman, gravida 1 para 0, presented to our hospital with repeated attacks of mild vaginal bleeding. Our patient, according to her sure reliable dates, was 16 weeks and three days pregnant. She had a history of primary infertility for seven years preceding this pregnancy, and she had sought medical advice for delayed conception one year after marriage. Male factor infertility was excluded by a single semen analysis and she was informed that her hysterosalpingography was quite normal; she was given medical treatment that she could not recall to improve her cycles (these were infrequent and irregular over the last five years). She used these medications for about four months after which she reported some improvement in her cycles but conception did not occur. A hormonal profile was ordered and our patient was examined transvaginally by ultrasound. Subsequently, she was informed she had bilateral polycystic ovarian syndrome. Her physician prescribed clomiphene citrate (Clomid, Global Napi, Egypt) 100 mg for five days beginning on the second day of her cycle. Our patient continued taking the drug without ovulation monitoring and the regimen was repeated with an increasing dose up to 200 mg daily for six successive months. Laparoscopic ovarian drilling was performed and our patient resumed using clomiphene citrate three months after the operation without prescription. Two months prior to admission, she took 250 mg of clomiphene citrate (five tablets per day) beginning from the second day onwards until the 13th day of the cycle.

**Figure 1:**
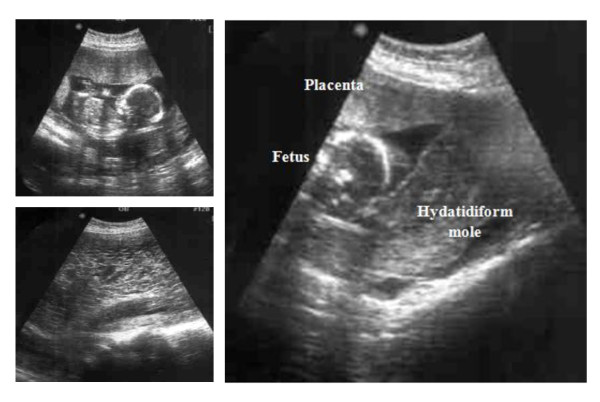
**Ultrasound picture of complete hydatidiform mole with coexisting fetus and placenta**.

Our patient had a missed period immediately after this haphazard regimen. Pregnancy was confirmed by a urine pregnancy test then by a trans-vaginal ultrasonographic examination that was carried out six weeks and three days after her last menstrual period. According to this ultrasound report, the gestational sac corresponded to seven weeks of gestation. However, there was no comment regarding the presence of theca lutein ovarian cysts. She did not follow-up with her pregnancy until she reached the 16th week of gestation. At that time, she experienced recurrent attacks of vaginal bleeding that she described as mild and dark colored. Apart from pallor and tachycardia, our patient appeared quite normal on general examination; her pulse was 106 beats/minute, blood pressure was 125/85 and her temperature was 37.2°C. Abdominally, her uterine fundal level was equivalent to 28 weeks and the uterus was dewy in consistency in most of its mass. A transabdominal ultrasound examination revealed a huge complete hydatidiform mole occupying the lower pole of the uterus and a coexisting fetus with its placenta that were enclosed within a separate sac (Figure 1). Our patient was admitted to our hospital and full laboratory investigations were ordered. Her blood test results revealed microcytic hypochromic anemia and her hemoglobin level was 9.6 g/dL. Other investigations were normal. In spite of her testing positive, her serum β-hCG level was relatively low for a patient with complete molar pregnancy (8354 mIU/mL and 7799 mIU/mL in two serum samples drawn two days apart). The technician did not suspect any technical error and he confirmed the accuracy of the result. However, we considered the possibility of falsely low results and accordingly, a senior specialist was consulted. She repeated the test with serial dilutions and β-hCG was found to be 1.876 million mIU/mL. Our patient was counseled about the risk of continuation of this pregnancy and the low possibility of fetal survival. However, our patient refused intervention and insisted on conservation.

**Figure 2:**
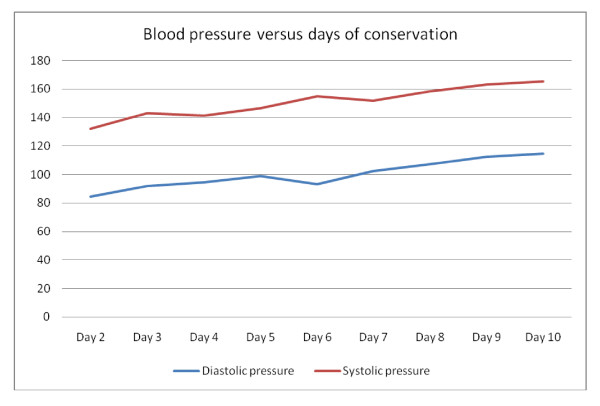
**Blood pressure follow-up mean readings during conservative management**.

The choice of conservative management was quite difficult; our patient was kept under strict observation and she was followed up using a four-hour interval chart for vital signs. Blood picture, renal chemistry (urea and creatinine), liver enzymes and thyroid function tests were ordered twice weekly, vaginal bleeding was observed and our patient was asked to report any pain, bleeding or other issues during the period of conservation. Our patient was offered genetic amniocentesis but she absolutely refused the procedure. On day 3, an increase in blood pressure above 140/90 mmHg was reported and it was found to be increasing (Figure 2). However, dipstick testing for albumin in her urine gave a negative result. Anti-hypertensive drugs were not given in order not to mask her actual blood pressure. She experienced palpitation, flushing and excessive sweating from day five and the thyroid function test was repeated; the results indicated thyrotoxicosis (T4 = 3.26 ng/dL, free T3 = 5.95 pg/mL, thyroid stimulating hormone (TSH) = 0.022 μIU/mL). Two attacks of mild vaginal bleeding were reported on days five and nine. The dipstick testing for albumin in urine became positive (1 plus) on day seven and the results became worse over the next few days reaching 4 plus on day 10. Our patient was recounseled about these unacceptable complications (thyrotoxicosis and severe pre-eclampsia) and for the low possibility of fetal survival. Again, the patient refused termination. Two days later, the patient developed a severe attack of vaginal bleeding; a hysterotomy was inevitable. She delivered a 680 g viable boy with no apparent congenital anomalies; the placenta was complete, about 15 cm in diameter and adjacent but identifiable from the coexisting molar pregnancy. The baby died half an hour later in the neonatal intensive care unit. A histopathological study confirmed the diagnosis of benign complete hydatidiform mole. Unfortunately, karyotyping was not available.

**Figure 3:**
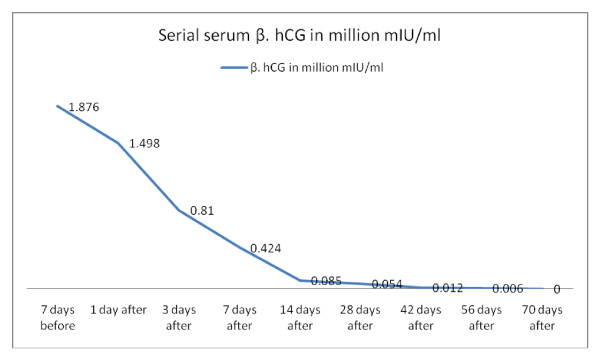
**Serum β-human chorionic gonadotropin (hCG) follow-up before and after termination of pregnancy after elimination of the 'high-dose hook effect'**.

Her serum β-hCG level was followed up after termination; it dropped progressively until it became negative after 70 days and remained so for 12 successive months (Figure 3). Serial dilution of the sample was no longer needed after termination.

## Discussion

Few cases of complete hydatidiform mole with a coexisting fetus have been reported over the last two decades. This broad term can be classified into three major types: (1) twin gestation in which one twin is a diploid fetus with a normal placenta (46 chromosomes, 23 maternal and 23 paternal) and the other twin is a complete hydatidiform mole (46 chromosomes of paternal origin) with no fetus (this is applied to our case report). (2) Singleton gestation consisting of a triploid fetus with partial hydatidiform mole placenta (69 chromosomes, 23 maternal and 46 paternal). (3) Twin gestation in which one twin is a diploid fetus with normal placenta (46 chromosomes, 23 maternal and 23 paternal) and the other twin is a triploid fetus with partial hydatidiform mole placenta (69 chromosomes, 23 maternal and 46 paternal) [4]. Categorization of the case is essential for proper management. Unlike partial hydatidiform mole that is commonly associated with multiple fetal anomalies and is managed by immediate termination of pregnancy [5], reported cases of twin pregnancy with complete hydatidiform mole (including our case) are not associated with fetal anomalies in the coexisting fetus; in some cases the mother has even given birth to fetuses that survived [6].

However, twin pregnancy with complete hydatidiform mole has a higher risk of maternal complications than partial hydatidiform moles; the same risk also applies to twin pregnancy with complete hydatidiform mole. These complications include early onset pre-eclampsia, thyrotoxicosis and persistent trophoblastic disease (PTD). Because of case rarity, the incidence of these complications cannot be exactly assessed in comparison with complete molar pregnancy; however, the incidence of PTD in reported case series varies from 19% to 50% [2,7]. The reason for this high incidence has not yet been clarified.

Accordingly, the management of these cases remains problematic: the fair possibility of fetal survival is weighed against the expected risk of maternal complications, and for this reason, many reported cases were managed by immediate termination. However, some authors support the option of conservation under strict hospital-based observation and follow-up. In this case, we support the latter policy for the following reasons. First, conservative management of some reported cases was successful, with fetal survival [5]. Second, most expected complications can be diagnosed by strict follow-up and clinical observation. Third, although the risk of PTD cannot be excluded during conservative management, this risk does not seem to increase with advanced gestational age [2]. According to these findings, we suggest that conservative management should always be a choice in such patients.

Amniocentesis is expected to be beneficial in decision making; a triploid fetus would be expected to be severely malformed and thus, termination of pregnancy would be recommended. A diploid fetus (46XX or 46XY of maternal and paternal origins) indicates a viable fetus with a normal placenta and therefore, pregnancy can be allowed to continue [4]. With regard to our patient, the option of amniocentesis was refused and we were obligated to manage our patient conservatively according to her will. Unfortunately, our patient experienced serious complications during conservation and hysterotomy was indicated; the fetus was alive but did not survive. However, as our patient was managed conservatively as long as possible and she did not experience any adverse post-operative outcomes including gestational trophoblastic disease, these findings may support the relative safety of conservation of these cases.

The falsely low result for serum β-hCG represents another interesting point in our patient's case; this is described as the 'high-dose hook effect'. This effect is not specific for β-hCG. It occurs when there is an inordinate amount of substance being measured by an immunoassay; this causes the formation of incomplete antibody-antigen complexes. Accordingly, below a certain threshold concentration, the assay will reflect the concentration of the substance correctly. Above this concentration, the assay will record falsely lower results as the concentration rises higher. This can be corrected by dilution of a sample and this was exactly what we did in this case [8].

It is infeasible to correlate between the occurrence of complete hydatidiform mole with a coexisting fetus and ovulation induction, particularly with clomiphene citrate, because the number of reported cases is limited. However, the association between complete hydatidiform mole and ovulation induction has not been confirmed; the incidence of complete hydatidiform mole following ovulation induction with clomiphene citrate was 1:659 [9,10]. However, Piura *et al. *reported 30 cases of twin pregnancy with complete hydatidiform mole and a co-existent fetus. Of these, nine cases (30%) were preceded by ovulation induction with either hMG/hCG (eight cases) or clomiphene citrate (one case). Five out of eight patients who received human menopausal gonadotropin (hMG)/human chorionic gonadotropin (hCG) were exposed to assisted reproductive technologies (*in vitro *fertilization/embryo transfer (IVF/ET) in three women, intra-cytoplasmic sperm injection/embryo transfer (ICSI-ET) in two women) [4]. This correlation may need further study and explanation.

## Conclusions

Twin pregnancy with complete hydatidiform mole is a rare condition that can be diagnosed by obstetric ultrasound examination. We cannot confirm or deny a correlation between this case and ovulation induction because the number of reported cases is still limited. Management of these patients is controversial. However, we suggest that conservative management is always a choice whenever strict hospital-based observation is available.

## Consent

Written informed consent was obtained from the patient for publication of this case report and any accompanying images. A copy of the written consent is available for review by the Editor-in-Chief of this journal.

## Competing interests

The authors declare that they have no competing interests.

## Authors' contributions

SAM participated in collecting medical data during patient follow-up and was a major contributor in writing the manuscript. MKA participated in collecting medical data during patient follow-up. AYA contributed to writing the manuscript. AAA reported the patient ultrasound that was illustrated in the manuscript. MMK contributed to writing the manuscript. NAM performed the related laboratory tests. All authors read and approved the final manuscript.
